# Serum visfatin in multiple sclerosis: distinct profiles in healthy controls, naive patients, and treated RRMS

**DOI:** 10.3389/fimmu.2025.1641260

**Published:** 2026-01-05

**Authors:** Saltanat Mert, İbrahim Acır, Hümeyra Öztürk Emre, Kübra Balcı, Edanur Demir

**Affiliations:** 1 Neurology Department, Başakşehir Çam and Sakura City Hospital, Istanbul, Türkiye; 2 Neurology Department, Bakırköy Dr. Sadi Konuk Training and Research Hospital, Istanbul, Türkiye

**Keywords:** adipokines, DMT, multiple sclerosis, NEDA-3, visfatin

## Abstract

**Aim:**

This study aimed to evaluate serum visfatin levels in treated patients with Relapsing-Remitting Multiple Sclerosis (RRMS) (PwMS) receiving disease-modifying therapy (DMT) and in naive MS patients (nMS), and to investigate their association with clinical characteristics, treatment status, and disease activity, including NEDA-3 status.

**Methods:**

A total of 45 PwMS under treatment at least 1 year, 20 nMS patients, and 44 age- and sex-matched healthy controls (HC) were included. Clinical and demographic data were recorded. Serum visfatin, lipid profiles, inflammatory markers, and vitamin levels were measured. Visfatin levels were compared not only between treated and naive MS patients but also within the treated group according to NEDA-3 status and treatment type (first-line vs. second-line disease-modifying therapy). Statistical analyses were performed to assess group differences and correlations.

**Results:**

Visfatin levels differed significantly across the three groups (Kruskal–Wallis H = 19.701, p < 0.001). Pairwise comparisons revealed significant differences between all groups: the control group had higher visfatin levels than both the nMS (p < 0.001) and PwMS (p = 0.006). The treated group also showed higher visfatin levels compared to the naive MS group (p = 0.014). Among PwMS visfatin levels were lower than in healthy controls (p = 0.006). Among PwMS patients, those meeting the NEDA-3 criteria had lower visfatin levels and EDSS scores compared with non-NEDA patients (p = 0.008 and p = 0.006, respectively). No significant correlation was found between visfatin and relapse count or EDSS. LDL and total cholesterol levels were significantly higher in patients receiving fingolimod.

**Conclusion:**

Across the three groups, visfatin levels differed significantly, with healthy controls showing the highest levels, followed by PwwMS, and the lowest levels observed in nMS patients. Lower serum visfatin levels in stable treated MS patients may reflect reduced inflammatory burden and effective immunomodulation. These findings suggest that visfatin could serve as a potential biomarker for treatment response in MS. Nevertheless, longitudinal studies including larger cohorts of naive patients are needed to clarify visfatin’s regulatory role in MS pathophysiology and its interaction with disease-modifying therapies.

## Introduction

Although the exact cause of multiple sclerosis (MS) is yet to be revealed, it is broadly accepted that the immune system has a pivotal role as far as the pathogenesis of the afore-mentioned condition is concerned. More specifically, this novel research in the field has emphasized the potentially significant contribution of adipose tissue in this context as it produces adipocytokines such as leptin, resistin, and adiponectin (1–3).

Visfatin has recently been identified as an adipokine synthesized in the brains of both animals and humans, produced by both adipocytes and macrophages, and expressed in various tissues, including the bone marrow, liver, kidneys, skeletal muscle, heart, lung muscle, adipose tissue, and immune cells such as lymphocytes, monocytes, and neutrophils (4, 5). As such, visfatin enhances the formation of proinflammatory cytokines, synthesizes adhesion molecules, and induces leukocyte activation (6, 7). Visfatin exerts its immunomodulatory effects primarily through the activation of NF-κB and MAPK signaling pathways, thereby enhancing the expression of costimulatory molecules and amplifying immune cell responses. Through these mechanisms, visfatin stimulates the production of proinflammatory cytokines such as IL-1β, TNF-α, and IL-6 in human leukocytes and monocytes. Consistently, it has also been shown to induce the synthesis of IL-1β, IL-6, IL-10, and TNF-α in dendritic cells and peripheral blood mononuclear cells. Collectively, these findings support the notion that visfatin functions as a potent proinflammatory adipocytokine (7–9).

Visfatin has been implicated in the pathophysiology of several disorders, including obesity, atherosclerosis, cardiovascular diseases, immune dysregulation, and ageing, primarily through its modulation of oxidative stress, apoptosis, and inflammatory pathways (10). Elevated circulating visfatin levels have also been reported in various inflammatory conditions such as acute ischemic stroke, migraine during attacks, chronic obstructive pulmonary disease, rheumatoid arthritis, and sepsis, where its concentration has been shown to correlate with inflammatory cytokines (11–15). In the context of MS, previous studies investigating adipokines have often included heterogeneous cohorts with variable or undocumented treatment exposure, which may contribute to inconsistent findings regarding adipokine dynamics (16).

The aim of this study was to evaluate serum visfatin levels in treated patients with relapsing remitting MS (RRMS), investigate their associations with demographic and clinical characteristics, and compare them with healthy controls. Considering that disease modifying therapies exert potent immunomodulatory and anti-inflammatory effects, we hypothesized that serum visfatin levels in treated RRMS patients might not follow the classical pro-inflammatory profile but rather reflect treatment efficacy and long-term disease control.

## Material and methods

The study population included 45 treated patients with RRMS (PwMS) patients receiving DMT, 20 naive MS patients (nMS), and 44 age- and sex-matched healthy controls; visfatin levels were compared across these three groups, and the patient cohort was further evaluated according to NEDA-3 status (NEDA vs. non-NEDA). Patients were recruited from the outpatient neurology clinic and were required to meet the 2017 revised McDonald diagnostic criteria for MS. All enrolled patients were under disease-modifying treatment at the time of inclusion.

Exclusion criteria comprised age below 18 years, a diagnosis of primary or secondary progressive MS, relapse within the preceding three months, and recent steroid exposure. Individuals with concomitant neurological, autoimmune, or systemic inflammatory conditions, as well as those with hypertension, diabetes mellitus, coronary artery disease, or active/chronic infections, were also excluded. Healthy controls were selected among hospital staff and volunteers with no history of neurological or systemic diseases and were matched individually to patients by age and sex.

The study protocol was approved by the local ethics committee (Approval No: E-96317027-514.10-260888486). All participants provided written informed consent after receiving detailed information about the study. The research was conducted in accordance with the principles of the Declaration of Helsinki.

This study recorded demographic data along with measurements of weight, height, body mass index (BMI), serum glucose, thyroid-stimulating hormone (TSH), aspartate aminotransferase (AST), alanine aminotransferase (ALT), creatinine, cholesterol, low-density lipoprotein (LDL), high-density lipoprotein (HDL), triglycerides, vitamin D, vitamin B12, high-sensitivity C-reactive protein (hs-CRP), and visfatin levels for all participants.

MS patients receiving DMT were categorized into two groups—those who met the NEDA-3 (No Evidence of Disease Activity) criteria and those who did not—and an additional group of 20 nMS patients was evaluated separately. Patients exhibiting no clinical relapses, no radiological activity, and no observed progression were considered to meet the NEDA-3 criteria. The treated patient group was further divided based on treatment into those receiving first-line DMT (teriflunomide or dimethyl fumarate) and those receiving second-line disease modifying treatments (fingolimod, ocrelizumab or natalizumab).

Venous blood samples were collected from all consenting participants following a 12-hour fast. These samples were taken between 8:00 and 10:00 am, drawn into serum separator tubes with gel, and immediately centrifuged to isolate the serum. The remaining part of the serum were kept at -80 °C prior to the analysis.

### Biochemical analyses

Fasting blood glucose, TSH, creatinine, LDL cholesterol, and routine inflammatory markers for all participants were measured using the Roche Cobas 8000 biochemistry and immunoassay systems available in the central laboratory.

### Visfatin analysis

The serum visfatin layers were quantified by means of a commercial enzyme-linked immunosorbent assay (ELISA) kit (Human Visfatin ELISA Kit) following the manufacturer’s protocol. All samples were tested in duplicate, and the intra-assay and inter-assay coefficients of variation for the ELISA kit were both below 10%, ensuring analytical reliability.

### Statistical analysis

The patient data collected in the study were analyzed using the IBM SPSS, version 30.0 (IBM Corp., Armonk, NY). Frequencies and percentages were reported for categorical variables, while mean, standard deviation, median, minimum, and maximum values were used as descriptive statistics for continuous variables. For group comparisons, visfatin levels were analyzed across three groups using the Kruskal–Wallis test. When overall significance was detected, pairwise comparisons were performed with Dunn–Bonferroni–corrected Mann–Whitney U tests. Continuous variables were summarized as median (IQR) if non-normal and compared using the Mann–Whitney U/Kruskal–Wallis tests with Dunn’s *post-hoc* correction; if normal, they were expressed as mean ± SD and compared with t-test/ANOVA. An approximate power analysis (n=45, α=0.05, two-tailed) indicated ~52% power for r=0.30, ~66% for r=0.35, and ~78% for r=0.40; therefore, negative correlations below the level of r<0.35 may have been missed. Associations were examined using Spearman correlation. Statistical significance was set at two-tailed p<0.05.

## Results

A total of 109 participants were included in the study, consisting of 45 PwMS patients (41.2%), 44 HC (40.3%) and 20 nMS (18.4%); however, the serum sample of one control subject could not be analyzed due to technical reasons. As shown in **Table 1**, there were no statistically significant differences between the treated PwMS and control groups in terms of age, sex, or BMI (p > 0.05).

**Table 1:** Distribution of demographic characteristics of the treated patients with MS (PwMS) and control groups.

Characteristics	Control (n=44)	PwMS (n=45)	p-value
Gender, n (%)			0.071
Male	24 (54.5)	15 (33.3)	
Female	20 (45.5)	30 (66.7)	
Age			0.139
Mean ± SD	32.0 ± 9.2	34.2 ± 8.8	
Median (Min-Max)	28 (18–60)	33 (18–50)	
BMI			0.110
Mean ± SD	24.3 ± 3.4	25.7 ± 4.7	
Median (Min-Max)	25.2 (17.5-30.9)	25.6 (17.6-39.4)	

Clinical characteristics of the treated patient group are presented in **Table 2**. The median disease duration was 3 years (range: 0.3–16 years). A total of 44.4% of patients had experienced at least one relapse in the past year, while 53.3% reported at least one relapse in the past two years. The mean Expanded Disability Status Scale (EDSS) score was 1.7 ± 0.7, with a median of 2. The most frequently used disease-modifying treatments were dimethyl fumarate (n = 17) and fingolimod (n = 18).

**Table 2:** The distribution of treatment findings in the PwMS group.

Variable	n (%)	Mean ± SD	Median (Min–Max)
EDSS		1.7 ± 0.7	2 (1–4)
Disease duration (years)		3.9 ± 3.5	3 (0.3–16)
≥1 relapse in the past 1 year	20 (44.4%)		
Number of relapses in 1 year		1.1 ± 0.3	1 (1–2)
≥1 relapse in the past 2 years	24 (53.3%)		
Number of relapses in 2 years		1.1 ± 0.3	1 (1–2)
Therapies			
Dimethyl fumarate	17 (37.8%)		
Fingolimod	18 (40.0%)		
Natalizumab	5 (11.1%)		
Ocrelizumab	3 (6.7%)		
Peginterferon	1 (2.2%)		
Teriflunomide	1 (2.2%)		

Laboratory findings are summarized in **Table 3**. Statistically significant differences were observed between the patient and control groups in terms of TSH (p = 0.047), creatinine (p = 0.023), vitamin D (p = 0.013), and visfatin levels (p = 0.006). Vitamin D levels were significantly higher in the patient group, whereas visfatin, TSH, and creatinine levels were higher in the control group. The mean visfatin level was 28.3 ± 27.2 ng/mL in PwMS group and 53.0 ± 43.8 ng/mL in the HC group. This difference was found to be statistically significant (p = 0.006).

**Table 3:** Distribution of laboratory measurements in the PwMS and control group.

Variables	Control (n=44)	PwMS (n=45)	p-value
Glucose			0.925
Mean ± SD	94.5 ± 23.5	92.3 ± 11.2	
Median (Min-Max)	91.0 (75–240)	91.0 (79–152)	
TSH			**0.047**
Mean ± SD	**2.6 ± 1.7**	**2.0 ± 1.0**	
Median (Min-Max)	**2.1 (1-11.1)**	**1.7 (0.7-5)**	
Creatinine			**0.023**
Mean ± SD	**0.8 ± 0.1**	**0.7 ± 0.1**	
Median (Min-Max)	**0.8 (0.5-1.1)**	**0.7 (0.5-1.1)**	
GFR			0.881
Mean ± SD	114.7 ± 11.3	115.1 ± 11.5	
Median (Min-Max)	114.0 (81–139)	117.0 (82–133)	
ALT			0.262
Mean ± SD	25.1 ± 26.4	23.8 ± 12.9	
Median (Min-Max)	18.5 (8–160)	21.0 (8–59)	
AST			0.241
Mean ± SD	20.3 ± 8.2	18.5 ± 6.1	
Median (Min-Max)	18.0 (11–60)	17.0 (10–38)	
Triglycerides			0.557
Mean ± SD	111.0 ± 63.3	102.3 ± 57.9	
Median (Min-Max)	95.5 (33–280)	82.0 (31–273)	
HDL			0.424
Mean ± SD	50.6 ± 13.6	52.9 ± 13.1	
Median (Min-Max)	48.0 (27–78)	52.0 (27–86)	
LDL			0.582
Mean ± SD	109.2 ± 34.1	112.4 ± 29.8	
Median (Min-Max)	104.5 (51–198)	106.0 (56–176)	
Total cholesterol			0.636
Mean ± SD	182.1 ± 37.5	185.7 ± 34.3	
Median (Min-Max)	182.5 (119–273)	180.0 (116–268)	
CRP			0.733
Mean ± SD	2.9 ± 4.6	4.1 ± 11.1	
Median (Min-Max)	1.7 (0.2-25)	1.7 (0.1-74.1)	
Vitamin D			**0.013**
Mean ± SD	**18.6 ± 9.8**	**27.2 ± 19.9**	
Median (Min-Max)	**18.0** (3–50)	**22.0** (5–127)	
Vitamin B12			0.202
Mean ± SD	326.4 ± 154.6	353.7 ± 137.9	
Median (Min-Max)	292.0 (24–865)	331.0 (141–859)	
Visfatin			**0.006**
Mean ± SD	**53.0 ± 43.8**	**28.3 ± 27.2**	
Median (Min-Max)	**32.2 (4.8-128)**	**21.5** (2–128)	

SD, Standard Deviation; GFR, Glomerular Filtration Rate; ALT, Alanine Aminotransferase; AST, Aspartate Aminotransferase; HDL, High-Density Lipoprotein; LDL, Low-Density Lipoprotein; CRP, C-reactive Protein; TSH, Thyroid-Stimulating Hormone; EDSS, Expanded Disability Status Scale. Bold values indicate statistically significant results (p < 0.05).

Correlation analysis among clinical variables in the patient group is shown in **Table 4**. A statistically significant, weak negative correlation was found between EDSS scores and the number of relapses over two years (r = –0.314; p = 0.035). No statistically significant correlation was observed between visfatin levels and either EDSS scores or relapse counts (p > 0.05).

**Table 4:** Distribution of the relationships between clinical measures in the PwMS group.

		Visfatin	1-Year Relapse	2-Year Relapse	EDSS
Visfatin	r	1.000	0.108	0.052	0.142
	p	–	0.479	0.733	0.352
	n	45	45	45	45
1-Year Relapse	r	0.108	1.000	**0.855**	-0.262
	p	0.479	–	**0.000**	0.082
	n	45	45	**45**	45
2-Year Relapse	r	0.052	**0.855**	1.000	**-0.314**
	p	0.733	**0.000**	–	**0.035**
	n	45	**45**	45	**45**
EDSS	r	0.142	-0.262	**-0.314**	1.000
	p	0.352	0.082	**0.035**	–
	n	45	45	45	45

r: correlation coefficient; p: probability value; n: number of patients. Bold values indicate statistically significant results (p < 0.05).

**Figure 1:**
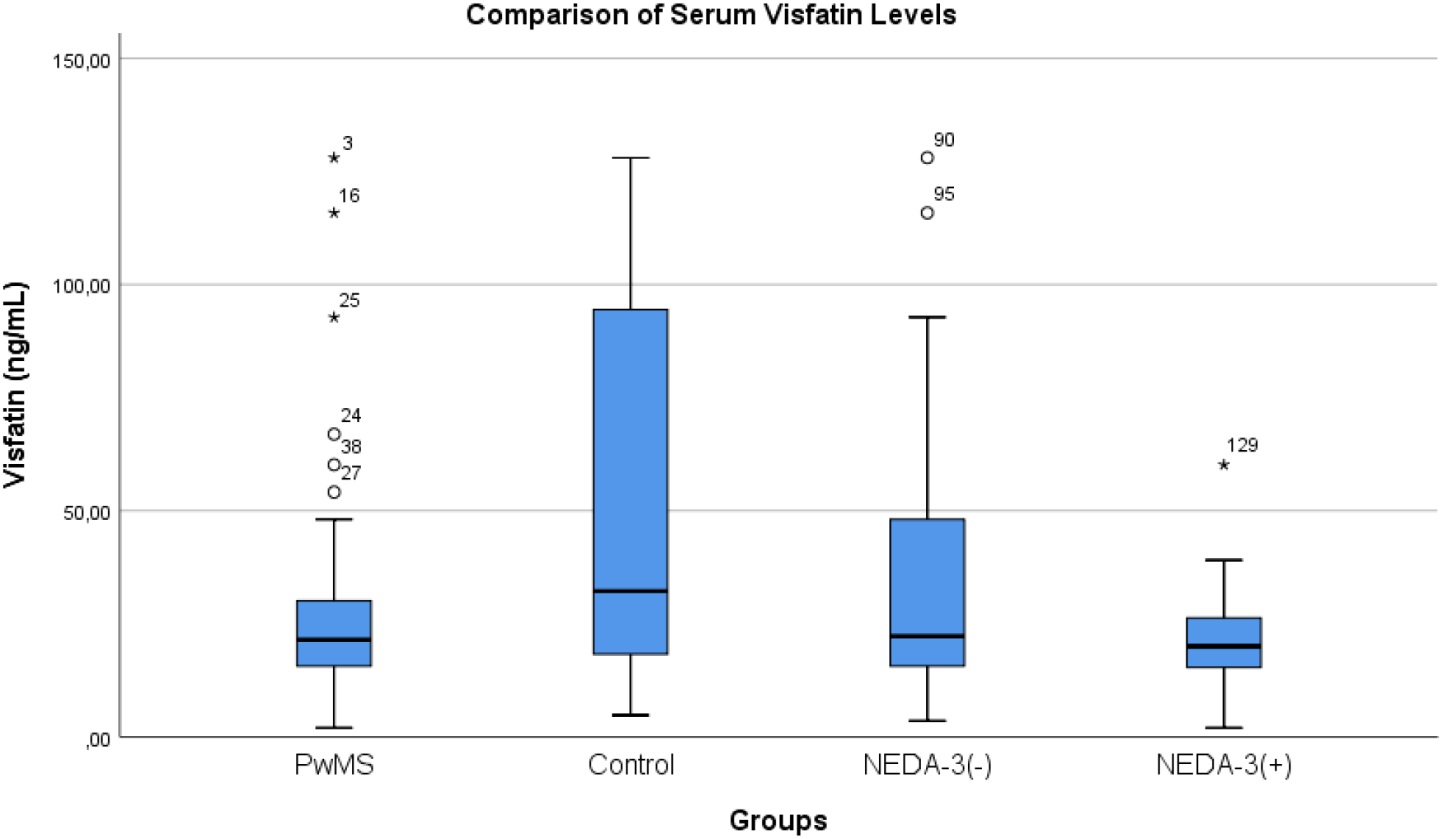
Comparison of serum visfatin levels between groups.

In the comparison between patients meeting and not meeting the NEDA-3 criteria, both the mean serum visfatin levels and the mean EDSS scores were significantly lower in the NEDA-3(+) group (17.3 ± 11.3 vs. 37.7 ± 33.1 ng/mL, p=0.008; 1.3 ± 0.4 vs. 1.7 ± 0.5, p=0.006). Additionally, hs-CRP levels were lower in the NEDA-3(+) group (1.2 ± 1.0 vs. 3.4 ± 3.9, p=0.015). No significant differences were observed between the two groups with respect to age, sex distribution, BMI, disease duration, vitamin D levels, or other laboratory parameters (**Table 5**). Treatment profiles differed between the groups, with second-line DMT use being more common in the NEDA-3 (–) group compared to NEDA-3(+), whereas first-line therapies predominated among NEDA-3(+) patients (p=0.049). When serum visfatin levels were evaluated across the entire cohort, the control group demonstrated a broader distribution and higher median values, while levels were lower and more homogeneous among PwMS. Within the patient subgroups, visfatin levels were lowest in the NEDA-3(+) group, followed by NEDA-3 (–), highlighting a more restricted distribution in patients with stable disease activity (**Figure 1**).

**Table 5:** Comparisons of demographic and clinical characteristics between PwMS groups based on NEDA-3 status.

Characteristics	NEDA-3 (–) (n = 24)	NEDA-3 (+) (n = 21)	*p* value
Age	32.04 ± 8.08	36.61 ± 9.06	0.083
Duration of disease (mean ± S.D, years)	2.98 ± 2.77	4.90 ± 4.04	0.770
EDSS	1.7 ± 0.5	1.3 ± 0.4	**0.006**
Height (cm)	166.8 ± 8.4	168.1 ± 8.2	0.601
Weight (kg)	71.0 ± 14.9	74.1 ± 17.4	0.528
BMI (kg/m^2^)	25.4 ± 4.7	26.0 ± 4.7	0.677
Visfatin (ng/mL)	37.7 ± 33.1	17.3 ± 11.3	**0.008**
Vitamin D (ng/mL)	26.6 ± 14.0	24.5 ± 12.1	0.580
hs-CRP	3.4 ± 3.9	1.2 ± 1.0	**0.015**
first-line DMT second-line DMT	15 (68.2%) 9 (39.1%)	7 (%31.8) 14 (60.9%)	**0.049**

DMT: disease modifying treatment. Bold values indicate statistically significant results (p < 0.05).

When the comparison including the 20 nMS patients was performed, visfatin levels also differed significantly across the three groups (Kruskal–Wallis H = 19.701, p < 0.001). Dunn–Bonferroni–corrected Mann–Whitney tests revealed significant differences in all pairwise comparisons. The HC group demonstrated significantly higher visfatin levels than both the nMS (p < 0.001) and PwMS (p = 0.006) groups. Additionally, the PwMS group exhibited higher visfatin levels compared with the nMS group (p = 0.014) (**Table 6**).

**Table 6:** Comparisons of visfatin levels among nMS, PwMS, and healthy control groups (Dunn–Bonferroni–corrected Mann–Whitney U tests).

Comparison	n	U Statistic	Z	p-value
nMS vs HC	20/44	156.000	-4.115	**0.000**
PwMS vs HC	45/44	656.000	-2.742	**0.006**
PwMS vs nMS	45/20	277.500	-2.452	**0.014**

n: number, nMS: naive MS, PwMS: treated patients with MS, HC: healthy control. Bold values indicate statistically significant results (p < 0.05).

## Discussion and conclusion

This study investigated the role of visfatin, a pleiotropic immunometabolic adipokine, in patients with MS. We found that serum visfatin levels were significantly lower in treated MS patients compared with healthy controls, and within the MS cohort, those meeting the NEDA-3 criteria exhibited reduced visfatin concentrations and EDSS scores compared with non-NEDA patients.

Visfatin has been described as a potent pro-inflammatory mediator, promoting preadipocyte differentiation and activating macrophages, monocytes, dendritic cells, neutrophils, and lymphocytes, thereby inducing cytokines such as IL-6, IL-1β, and TNF-α (7, 17–21). It also activates NF-κB signaling in vascular endothelial cells and has been linked to neuroinflammatory conditions such as migraine, where plasma levels rise during attacks (12, 17). Elevated visfatin has additionally been reported in systemic inflammatory and metabolic disorders, including metabolic syndrome and acute myocardial infarction, in which high levels predicted poor outcomes (22, 23). Similarly, Kadoglou et al. found increased visfatin in acute ischemic stroke and carotid artery stenosis, correlating with CRP as a marker of systemic inflammation (24).

Against this background, the reduced visfatin levels observed in our MS cohort, particularly in patients achieving NEDA-3, are unlikely to be coincidental. Unlike treatment-naive populations, all patients in this study were receiving DMTs with strong immunomodulatory and anti-inflammatory properties. Suppression of systemic inflammatory mediators by DMTs likely contributed to diminished visfatin levels, consistent with the concept that therapeutic modulation of immune activity can reduce adipokine-driven inflammation. Thus, while earlier studies reported visfatin elevation in untreated or highly inflammatory disease states, our results support the view that effective disease control under DMT parallels lower circulating visfatin. The association with NEDA-3 may therefore reflect both intrinsic disease quiescence and treatment intensity.

This interpretation is supported by prior evidence that DMTs reshape adipokine profiles. For example, significant changes in adiponectin and FABP-4 have been observed under dimethyl fumarate, and distinct adipokine signatures have been reported with second-line therapies. Leptin, another adipokine with immunomodulatory activity, has been shown to increase after interferon beta-1a therapy (1, 25–27), whereas adiponectin—an anti-inflammatory mediator—rises following dimethyl fumarate treatment (28). Moreover, natalizumab and fingolimod have been associated with therapy-specific alterations in circulating adipokines (29). Collectively, these data reinforce the concept that adipokines, including visfatin, may serve as biomarkers of therapeutic response rather than markers of disease activity alone.

Our finding of reduced visfatin levels in clinically stable PwMS aligns with this framework. Adipokines such as leptin, resistin, and chemerin (generally elevated in MS), and adiponectin (reduced in MS), contribute to pathogenesis via T-cell polarization and blood–brain barrier integrity. DMTs may indirectly modulate these axes, while metabolic factors such as BMI, lipid status, and glycemic control can further influence adipokine biology. Recent reviews have emphasized how adipokines integrate metabolic and immune pathways in MS and highlighted their modulation through both lifestyle and pharmacologic interventions (16, 30). Within this context, our data suggest that visfatin may act as an index of immunometabolic tone in treated, clinically stable MS patients.

An additional consideration is the compartment-specific behavior of visfatin. Discrepancies between serum and CSF cytokine levels are well documented, raising the possibility that serum reductions may not reflect CSF or lesion-specific activity (31, 32) Visfatin could theoretically remain elevated within the CNS, even when peripheral levels are suppressed. Future studies incorporating CSF analyses, progressive MS cohorts, and longitudinal follow-up are needed to clarify compartmentalized roles of visfatin in MS pathophysiology.

We also observed high variability in visfatin concentrations within both PwMS and control groups (28.3 ± 27.2 ng/mL vs. 53.0 ± 43.8 ng/mL), with standard deviations nearly matching mean values. This marked inter-individual heterogeneity may reflect differences in metabolic status, adiposity, or treatment response. Although our analyses identified statistically significant differences, this dispersion reduces the interpretability of absolute thresholds, underscoring the need for larger, stratified studies.

Our study demonstrated that visfatin levels differed markedly among HC, nMS, and PwMS groups. The stepwise pattern identified in the three-group comparison (HC > PwMS > nMS) is thought to represent an important immunometabolic signal in the context of MS pathophysiology. The finding that nMS patients exhibited the lowest visfatin concentrations suggests that active inflammation and metabolic stress may exert a suppressive effect on visfatin production or its circulating levels. Visfatin represents the circulating form of NAMPT, the rate-limiting enzyme in NAD^+^ biosynthesis, and plays a critical role in immune cell metabolism, energy homeostasis, and regulation of the inflammatory response (33). Accordingly, increased cellular energy demand and immune dysregulation in active MS may contribute to decreased circulating visfatin levels. The observation that treated patients displayed significantly higher visfatin levels than the nMS group—yet still lower than healthy individuals—indicates that DMTs may partially normalize the immunometabolic axis without fully restoring it. The pronounced reduction observed in both PwMS and nMS groups suggests that visfatin may serve as a potential biomarker for early or active disease states. The partial elevation seen in patients undergoing treatment further supports the possibility that visfatin could act as an indirect indicator of DMT response.

Beyond visfatin, our findings also revealed higher LDL and cholesterol levels in patients treated with fingolimod compared with those on other therapies. This observation aligns with prior reports linking fingolimod to hyperlipidemia and is clinically relevant given the increased cardiovascular risk in PwMS, which may be amplified by DMT side effects, corticosteroid exposure, and reduced physical activity (34–37). Hyperlipidemia itself has been associated with worse disability progression and higher cardiovascular mortality, suggesting that metabolic monitoring should be integrated into long-term MS care.

This study has several limitations. First, only RRMS patients were included, limiting generalizability to progressive MS. Second, visfatin was measured exclusively in serum, without CSF or tissue samples, preventing compartment-specific analysis Finally, we lacked access to postmortem brain tissue for immunohistochemistry, which could further clarify visfatin localization in MS lesions.

In conclusion, this study demonstrates that visfatin levels differ significantly among healthy controls, naive MS patients, and treated PwMS, exhibiting a stepwise pattern (HC > PwMS > nMS) that reflects both disease activity and treatment-related immunometabolic modulation. Lower visfatin levels in naive and non-NEDA patients, together with partially restored levels in treated individuals, support the role of visfatin as a potential biomarker of early disease activity and therapeutic response. The association of fingolimod with higher LDL and cholesterol levels further highlights the relevance of integrating metabolic monitoring into MS management. While these findings contribute to understanding the immunometabolic landscape of MS, longitudinal studies including progressive phenotypes, CSF analyses, and tissue-based investigations are needed to clarify visfatin’s compartment-specific dynamics and its potential utility as a clinically meaningful biomarker.

## Data Availability

The raw data supporting the conclusions of this article will be made available by the authors, without undue reservation.
